# Cognitive styles and future depressed mood in early adulthood: The importance of global attributions

**DOI:** 10.1016/j.jad.2014.08.057

**Published:** 2015-01-15

**Authors:** R.M. Pearson, J. Heron, K. Button, R.P. Bentall, C. Fernyhough, L. Mahedy, L. Bowes, G. Lewis

**Affiliations:** a School of Social and Community Medicine, University of Bristol, UK; b Institute of psychology health and society, University of Liverpool, UK; c Department of Psychology, Durham University, UK; d Institute of Psychological Medicine and Clinical Neuroscience, School of Medicine, Cardiff University, UK; e Department of Experimental Psychology, University of Oxford, UK; f Division of Psychiatry, UCL, UK

**Keywords:** ALSPAC, Depression, Cognitive styles, Latent traits, Global attribution

## Abstract

**Background:**

Cognitive theories of depression suggest that beliefs of low self-worth and the tendency to attribute negative events to causes that are global (widespread rather than specific) and stable (will persist rather than change in the future) are associated with the development of depressed mood. Such theories are supported by evidence from prospective studies and have guided the development of successful treatment and prevention strategies such as CBT. However, the relative importance of different psychological constructs within cognitive theories is unknown. This is important to refine cognitive theories and develop more efficient prevention strategies.

**Method:**

We used prospective data from over 3500 young adults from the Avon Longitudinal Study for Parents and Children (ALSPAC) cohort in the UK to investigate the association between cognitive style, measured by short forms of the Dysfunctional Attitudes Scale (DAS) and Cognitive Styles Questionnaire-Short Form (CSQ-SF) at age 18, and future depressed mood at age 19. Structural equation modelling techniques were used to separate cognitive style constructs.

**Results:**

Cognitive styles were associated with future depressed mood, independently of baseline mood, both as measured by the DAS-SF and the CSQ-SF. Of the different CSQ-SF constructs, only global attributions were associated with both baseline and future mood independently of other constructs.

**Limitations:**

The study was subject to attrition and the follow-up was relatively short (10 months).

**Conclusion:**

The findings suggest that the tendency to attribute negative events specifically to global causes could be particularly important for depression. Reducing global attributions is potentially important in the prevention and treatment of depression.

## 1 Introduction

Depression during adulthood is one of the leading causes of disabilities worldwide. It is associated with substantial disruption to social, educational and occupational functioning (Thapar et al., 2012). Prevention and treatment of depression are therefore a priority for psychiatry research.

Cognitive Behavioural Therapy (CBT), which is based on cognitive theories of depression, can be effective in treating and preventing depression (Churchill and Hunot, 2001, Butler and Chapman, 2006, Brugha and Morrell, 2011). However, CBT does not work for everyone and is time consuming, specialist and expensive (Button et al., 2012). Further refinement of the cognitive theories on which it is based is therefore important to guide improvements in the efficiency and efficacy of CBT and other psychological approaches, in prevention and treatment. For example, there are several related psychological ‘constructs’ outlined in cognitive theories of depression and used in CBT. Currently there is no agreed emphasis on any one construct over others. Identifying which constructs are most important to depression could inform a prevention strategy that is more efficient and achievable than unnecessarily tackling all the constructs outlined in cognitive theories.

A psychological ‘construct’ may be thought of as a variable which is not directly observable but which can be inferred from one or more measure of ‘manifest’ variables. For example, intelligence itself is not directly observable; to measure it we rely on its manifestation in observable information such IQ test scores. The same applies to cognitive constructs relevant to depression. One important construct outlined in Beck׳s cognitive theory of depression is a permanent ‘negative’ self-belief system, most commonly measured by the Dysfunctional Attitudes Scale (DAS) (Weissman, 1979, Beck, 2005). In their similar ‘hopelessness theory of depression’, Alloy and Abramson emphasise the importance of ‘attributional style’. Across the several reformulations of their theories, they outline, amongst others, four constructs related to attribution of adverse events which they propose are ‘depressogenic’: attribution of adverse events to causes that impact *self-worth*׳ (means we are flawed in some may) and that are *internal* (our fault), *global* (impacts all aspects of our world) and *stable* (will impact our future) (Alloy et al., 1988). Cognitive theories also suggest that these ‘depressogenic’ attributions tend to be made across all scenarios faced by individuals. Although, of course, the attribution made about a particular event will, to some extent, be determined by the character of the event. These attributions are most recently measured using the Cognitive Styles Questionnaire (CSQ) (Haeffel and Gibb, 2008, Meins and McCarthy-Jones, 2012).

There are commonalities between the negative belief systems measured by the DAS and attributional styles. For example, we draw upon our belief systems to explain the world around us: if we believe we are flawed, when something bad happens to us we attribute it to such flaws (Bentall et al., 2001). Therefore, measuring the use of beliefs in interpretation of events (i.e., CSQ) is likely to also measure the presence of negative beliefs (i.e., DAS score). Consistent with this, previous work reports that there is no longer evidence for an association between DAS scores and depression once CSQ scores are accounted for (Haffel et al., 2005). In contrast, higher CSQ scores were associated with depression irrespective of DAS scores, suggesting that there are constructs from the CSQ that uniquely contribute to depression. However, this study was cross-sectional and explored a retrospective association with depression. Therefore, it did not indicate whether the DAS or CSQ was associated with future depression or indicate which constructs of the CSQ were uniquely important.

A few studies have investigated the *prospective* association between the CSQ and/or the DAS and later depression. Several of these studies report evidence for an association with future depression (Metalsky and Joiner, 1992, Zuroff and Mongrain, 2004, Alloy and Abramson, 2006, Hankin, 2008). However, these studies were relatively small (*N*<400). In larger low-risk samples results are less consistent: some report little evidence for a main association between cognitive style and later depression (Lewinsohn et al., 2001), whilst another large community-based female sample found an association between the DAS and future depressed mood (Evans et al., 2005). Further longitudinal studies with large sample sizes are required to help resolve this inconsistency and identify whether associations between cognitive styles and depression are questionnaire- (CSQ or DAS) or construct-specific.

Few studies have compared different cognitive style constructs and those that do report no differences in the association between construct sub-scales and future depression (Hankin and Abramson, 2001). However, previous studies compared cognitive style constructs using observed sub-scale scores which may not adequately separate constructs. This is because such an approach does not allow for the fact that the observed scores are only a manifestation of the underlying hypothetical construct of interest and thus contain measurement error. In contrast, factor analytic techniques such as those employed in structural equation modelling (SEM) are based on the assumption that observed information reflects an unobservable construct and measurement error is accounted for (Kline, 2011). In addition to measurement error there are further sources of ‘nuisance’ variance which are accounted for by SEM. In the case of the CSQ, each construct of interest is measured in response to several different scenarios, such as ‘getting on badly with your parents’ or ‘having a bad job evaluation’. According to the cognitive theory of depression cognitive style constructs should be elicited across scenarios; however, in a test setting, these different scenarios may introduce further ‘nuisance’ variance if some scenarios are more salient to certain participants than others.

If these sources of ‘nuisance’ variance are not accounted for (as in observed summed scores) any ‘true’ differences between constructs may be masked. For example, if all observed global attribution item scores are summed up, scores represent: 1) true differences in the individual׳s global attribution construct, 2) measurement error or 3) differences in the salience of the scenarios to individuals. By deriving construct specific latent traits, SEM separates the construct variance from the other two ‘nuisance’ sources (see Section 2).

In the present study we used SEM techniques to model prospective data on cognitive styles measured by short forms of the DAS and CSQ at age 18 and depressed mood at both age 18 and 19 from over 3500 young adults from the ALSPAC cohort in the UK. We have previously reported a positive cross-sectional association between CSQ scores and depression in this sample (Pearson et al., 2013); however, the prospective association with future depression and the relative contribution of different constructs were not investigated. Thus, in this study we investigated the following questions:

1. Is there an association between cognitive styles and later depressed mood (both continuous scores and categorical depressed/not depressed), having adjusted for baseline depressed mood?
2. Which specific cognitive style constructs (separated using latent traits) are independently associated with later depression? This may enable refinement of the cognitive theory of depression using empirical data.

## 2 Method

### 2.1 Sample

The sample comprised participants from the Avon Longitudinal Study of Parents and Children (ALSPAC). All pregnant women resident in the former Avon Health Authority in south-west England, having an estimated date of delivery between 1 April 1991 and 31 December 1992, were invited to take part. The children of 15,247 pregnancies were recruited (Boyd and Golding, 2013, Fraser and Macdonald-Wallis, 2013). Ethical approval for the study was obtained from the ALSPAC Law and Ethics Committee and the Local Research Ethics Committees. More detailed information on the ALSPAC study is available at http://www.alspac.bris.ac.uk. Detailed information has been collected on the cohort, including regular self-reported information from mothers and children and face-to-face assessments in research clinics. The present study reports data from the sample of ALSPAC offspring who attended the most recent research clinics for the children at age 18, where CSQ and DAS measures were taken.

Our starting sample was those with complete primary exposure (cognitive style measures) data (*N*=3845). A sample of 1698 also had data for depressed mood (outcome) at both 18 and 19. However, missing depression data was imputed and all analyses described below were repeated using the same starting sample size (*N*=3845) in sensitivity analyses.

### 2.2 Measures

#### 2.2.1 Cognitive Styles Questionnaire-Short Form (CSQ-SF)

A short version of the Cognitive Style Questionnaire (CSQ) developed from the original Cognitive Style Questionnaire was administered to the ALSPAC children at the fourth Teen Focus clinic (age 18). The CSQ is a frequently employed measure of negative cognitive style in relation to depression. The reliability and validity of the short version (CSQ-SF) have been established in young adults (Meins et al., 2012). The short version focuses on 8 negative hypothetical events relating to failures in academic, employment and interpersonal relationships. For each event participants are told to vividly imagine themselves in that situation and think carefully about the reason for the event. Participants then rate the extent to which this reason was caused by internal vs. external factors (caused by themselves or others), specific vs. global factors (the cause will impact all areas of life or just this specific situation) and stable vs. unstable factors (the cause will persist and lead to the same outcome in the future), and to which it reflects their self-worth (means they are flawed), on a Likert scale of agreement from 1–5. A fifth construct relating to negative consequences was omitted from the CSQ-SF. For each scenario, there are two items related to each of the 4 constructs resulting in 8 items across 8 scenarios (64 items in total). Total scores could range from 64 to 320 and subscale scores could range from 16 to 80. Higher scores indicate a more ‘negative’ style. Total scores and subscale scores were computed and showed normal distributions. Internal consistency for the total score in the current sample was *α*=0.88 which is comparable to previous studies (Meins et al., 2012). Internal consistency for sub-scale items was: stable, *α*=0.74; self-worth scale, *α*=0.84; global, *α*=0.69, and internal, *α*=0.56. Due to this low internal consistency, poor factor loadings and low correlations with other sub-scales and the DAS-SF, the internal sub-scale was not included in analyses.

#### 2.2.2 Dysfunctional Attitudes Scale-Short Form (DAS-SF)

The DAS-SF was also administered at the focus clinic. The DAS-SF is a self-report questionnaire containing nine items taken from the original Dysfunctional Attitude Scale (Weissman, 1979), using item-response analysis to provide an efficient and accurate assessment of dysfunctional attitudes (Beevers et al., 2007). The DAS is designed to assess Beck׳s ‘negative’ self-belief construct. Subjects rated their agreement to each of the 9 statements on a Likert scale of agreement from 1 to 5. Scores could range from 9 to 45, with higher scores reflecting more dysfunctional attitudes. The DAS-SF has reliability and validity in both student and patient samples (Beevers et al., 2007). Internal consistency of the total score in the current sample was *α*=0.80.

#### 2.2.3 Depression at age 18 and 19

##### 2.2.3.1 Mood and Feelings Questionnaire-Short version (SMFQ)

The SMFQ was also administered at the clinic at age 18 and again approximately 10 months later in a postal questionnaire (age 19). The SMFQ is a brief measure of depressive symptoms designed for children and adolescents aged 6–18 years (Angold et al., 2002). The 13-item SMFQ consists of statements relating to the occurrence of low mood (e.g. “I felt miserable or unhappy”). The respondent is asked to rate each statement (for the past two weeks) as ‘not true’, ‘sometimes true’, or ‘true’ (score 0–2 respectively). The scores on each individual item are summed, producing a total score ranging from 0 to 26. The total score may be dichotomised in order to classify individuals as depressed or not-depressed. In the present study we took a cut-off point of 10/11, which has previously been shown to have a high sensitivity and specificity (Thapar and McGuffin, 1998). In the current sample the SMFQ has been validated against a computerised clinical interview which derives diagnoses of depression according to ICD-10 criteria (see below). The discriminatory ability of the SMFQ for depression diagnosis was very high (area under ROC curve 0.90) (Turner et al., 2014). In regression models the binary SMFQ variable was primarily used because the SMFQ is not normally distributed. However, in the latent trait analyses a continuous latent trait for SMFQ was used to take full account of variation in symptoms.

##### 2.2.3.2 Clinical Interview Schedule-Revised (CIS-R)

Depression in the offspring at 18 was also measured using the computerised version of the Clinical Interview Schedule-Revised (CIS-R). The CIS-R is a computerised interview which derives a diagnosis of depression according to ICD-10 criteria. The CIS-R is designed for, and has been widely used within, community samples including in the National Surveys of Psychiatric Morbidity and the 1958 birth cohort (Lewis and Pelosi, 1988, Bebbington and Brugha, 2000). A binary variable indicating a primary diagnosis of major depression on the CIS-R or no such diagnosis was used to remove those with a diagnosis of depression at 18 from analysis.

#### 2.2.4 Confounding variables

Maternal age at the index child׳s birth (in years), social class (1–5) and child sex were recorded at the beginning of the ALSPAC data collection and these variables were included as covariates. Adjusting for baseline depressed mood should also provide a further proxy for shared risk variables.

### 2.3 Analysis

#### 2.3.1 Summed score approach

In the first stage of the analysis we investigated the cross-sectional and prospective associations between CSQ-SF total and sub-scale scores and DAS-SF total scores with SMFQ binary outcomes (depressed/not depressed) at age 19, using logistic regression analyses in Stata version 12. Baseline depression was controlled for in two ways: 1) to account for clinical depression, those with a diagnosis of depression at 18 using the CIS-R were removed and 2) to account for sub-threshold symptoms we adjusted for SMFQ total scores at age 18.

We imputed for missing data because a complete case analytical approach can lead to biased results if the data are not missing completely at random. As with all cohort studies, ALSPAC has suffered from substantial dropout over the last two decades. However, we are confident in our ability to build an adequate imputation model for these missing data due to the wealth of *auxiliary* measures that can be employed for this purpose. For example, given that there is substantial information on socio-demographic variables in ALSPAC which predict missingness, missing information can be assumed dependent on observed data (missing at random assumption). We employed a fully conditional specification as implemented in the MICE algorithm in STATA 12 using all variables described in the analyses and additional socio-demographic indicators of missingness (list available on request) to predict missing data across 100 imputed datasets. Monte Carlo errors were less than 10% of the standard error and FMI values were no larger than 0.8. Earlier measures of child depression and neuroticism were used to predict later depression allowing imputation to those with complete exposure data (*n*=3845) (White et al., 2011). The imputation method is based on regression equations to predict the missing variable. Therefore, the unique associations between *each* imputed variable and the predictor variables are used and every imputed variable is imputed using a unique set of regression equations.

In order to investigate the potential impact of missing exposure data, where strong predictors of cognitive styles (depressed mood and neuroticism measures) were available we extended sensitivity analysis to a larger sample which was more representative of the original ALSPAC cohort (*N*=8446). Analyses were conducted post-imputation by combining estimates across imputed data sets using Rubin׳s rules (White et al., 2011).

#### 2.3.2 Latent-trait approach

In the next stage of analysis we investigated the association between separate constructs of the CSQ-SF and depressed mood at both 18 and 19, using structural equation modelling techniques in order to separate ‘of interest’ variance from ‘nuisance’ variance. As described in Section 1, variance unrelated to our hypothesis, or ‘nuisance’ variance, arises from two sources: scenario-specific method variance and further unexplained variance likely to reflect measurement error. Using latent traits, however, nuisance variance (i.e., variance common to scenarios or unexplained variance likely to represent measurement error) is accounted for. This is because the latent trait for each construct of interest represents only information from the questionnaire items that is both *common* and *unique* to that latent trait (e.g., only variance common and unique to global attribution items forms the global attribution latent trait).

**Fig. 1:**
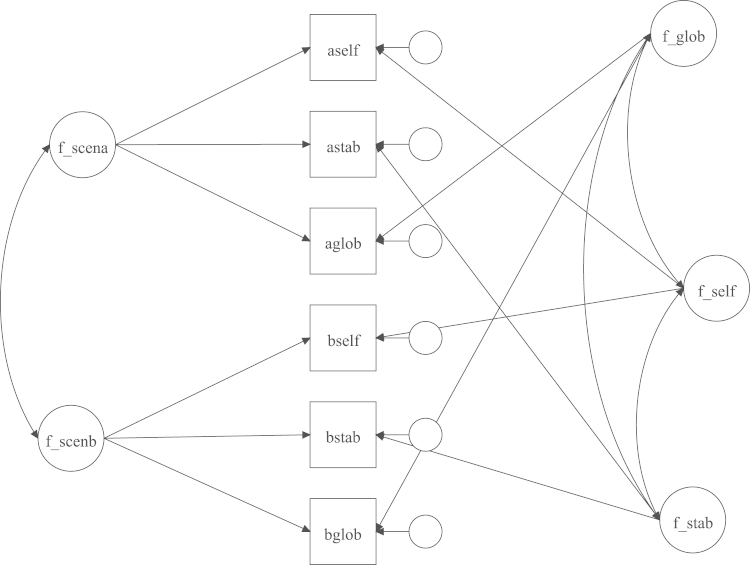
Factor structure of the CSQ-SF. The figure shows the structure across two of 8 scenarios.

Latent factors for each construct of the CSQ-SF were derived using confirmatory factor analysis (CFA) in M*plus* version 7. Construct-specific items (sum of 2 items relating to each construct from each scenario) were loaded on to ‘construct’ factors. There were 3 construct factors: global (f_glob), stable (f_stab) and self-worth (f_self) each derived from 8 items, one for each scenario. Scenario-specific variance was accounted for by deriving ‘scenario’ factors; items also loaded on to a factor representing the scenario within which they appeared. Each item thus cross-loaded on to one ‘construct’ and one ‘scenario’ factor (see Fig. 1). As scenario-specific variance was accounted for in this way, construct factors represent *construct*-specific variance *across* the 8 scenarios. Loadings for construct items on to construct factors ranged from 0.2 to 0.6 (note these are cross-loadings). Separate latent factors for SMFQ scores at both time points were also derived from CFA analyses loading all 13 items onto a single factor.

In assessing the fit of the CFA model, three model fit statistics were used: 1) root-mean-square error of approximations (RMSEA; Steiger, 2004); 2) comparative fit index (CFI; Hu et al., 1992); and 3) Tucker–Lewis fit index (TLI; Tucker and Lewis, 1973). Goodness of fit was determined in accordance with (Hu et al., 1992) and was indicated by CFI and TLI values of over 0.95, and RMSEA of less than 0.06. Multiple indices were used as they provide a more comprehensive evaluation of model fit. Model fit for this CSQ factor model (with method factors modelled) was good: CFI 0.973; TLI 0.962; RMSEA 0.030 (90% CI 0.027–0.033).

For each CSQ-SF item, factor loadings, factor variances and residual variances were used to calculate the proportion of variance which was explained by the construct factor (‘of interest’), the scenario factor and residual variance (in combination: ‘nuisance variance’). These are presented in Table E1. On average 23.4% of items׳ variance was ‘of interest’ and formed the separate construct factors for global, self-worth, and stable attributions, highlighting that sum scores for each construct include a high proportion (76.7%) of nuisance variance.

We then used these latent factors in a series of linear regression models to investigate the association between each construct factor and SMFQ factors at a) age 18, b) age 19 and c) age 19 adjusted for SMFQ scores at age 18. These associations were investigated initially in a combined model (each construct factor mutually adjusted for other construct factors) using a maximum likelihood estimator. In order to look at the association between SMFQ and each construct factor in isolation, plausible values (http://www.statmodel.com/download/Plausible.pdf) for each construct factor were imputed using a Bayesian estimator across 50 datasets. Estimated values for each construct factor across these 50 models were then used to investigate the association between each construct factor and SMFQ in *separate* models.

## 3 Results

### 3.1 Descriptive statistics

Sample demographics for the sample of adolescents who comprised our complete case sample as compared with the rest of ALSPAC are reported elsewhere (Pearson et al., 2013). Adolescents with complete cognitive style data came from more socially advantaged families. Descriptive statistics according to cognitive styles score are presented in Table 1, Table 2.

### 3.2 Main Analyses

#### 3.2.1 Summed score approach

##### 3.2.1.1 Association between CSQ-SF and DAS-SF and depressed mood at 18 and 19

As can be seen in Table 3 there was evidence that both CSQ-SF and DAS-SF were associated with later depressed mood independently of baseline mood. There was little evidence that the association with DAS-SF was independent of CSQ-SF, while the association with the CSQ-SF was relatively unaffected by adjusting for the DAS-SF. There was little evidence of any specific effects according to constructs of the CSQ-SF using observed sum scores. Indeed, including all CSQ-SF subscale summed-scores in the same mutually adjusted regression model resulted in no evidence for independent associations between depressed mood at 19 (also adjusted for baseline mood) and any of the CSQ-SF construct summed scores (in the combined model the OR for each 1 sd increase in global attributions was 1.16 [0.95–1.42, *P*=0.133] for stable attributions 1.02 [0.82–1.26, *P*=0.820] and self-worth attributions 1.16 [0.95–1.41, *P*=0.153]).

##### 3.2.1.2 Missing data sensitivity analyses

As can be seen in Table 4, results were comparable following imputation for missing data up to a starting sample with complete exposure data (Model 5, *n*=3845). In contrast, once this was extended to a larger sample (*n*=8446) many of the independent associations were diminished, potentially indicating that these associations were over-estimated in the complete case sample. However, there was remaining evidence that the global attribution sub-scale was independently associated with future depressed mood.

#### 3.2.2 Latent-trait approach

As can be seen in Table 4, there was also evidence for an association between stable attributions and later depression that was independent of baseline depression, but this association was not independent of other construct factors. There was no evidence for an association between self-worth and later depression once baseline depression or other constructs were accounted for. In contrast, there was a robust association between *global* attributions and later depressed mood, independently of baseline depressed mood and other CSQ-SF constructs. Therefore, the conclusion of this analysis is that global attributions are the only construct to be independently associated with depression; other constructs did not appear to explain any unique variance over and above that explained by global attributions.

## 4 Discussion

The findings provide evidence that cognitive style is associated with future depressed mood independently of baseline mood, both as measured by the DAS-SF and the CSQ-SF. However, there was little evidence that dysfunctional attitude scores (DAS-SF) were associated with later depression after adjusting for CSQ-SF scores. In contrast, the association between the CSQ-SF and depression was unaffected by including the DAS-SF. This is consistent with theoretical models (as described in Section 1) and with previous work (Haeffel et al., 2008). It suggests that the CSQ-SF captures the depression relevant constructs also measured on the DAS-SF. Of the CSQ-SF *latent* constructs, global attributions were the only construct to be associated with both baseline and future mood independently of other constructs.

Global attributions reflect the tendency to believe that causes of negative events are widespread and influence not only this situation but all other aspects of one׳s life, including those unrelated to the event in question. The tendency to make global attributions may also reflect the construct of ‘conditional beliefs’ outlined by Beck. If someone tends to always think that a negative event is caused by a characteristic that will affect all other areas of their life, this suggests they have a belief system that if one thing goes wrong then lots of other things will or there is something wrong with them that will affect all other things (see case example). Observational studies alone do not provide causal evidence. However, the findings suggest that attributing negative events to such global causes could be important to the development of later depression, irrespective of whether the cause is also perceived as stable and reflecting low self-worth. In contrast, in the absence of a global attribution to the cause, attribution of negative life events to causes which are stable and reflect low self-worth may not be sufficient to lead to future depression.

### 4.1 Strengths and limitations

There are several strengths of the current study. To our knowledge this is the largest sample to date in which prospective associations between measures of cognitive styles and later depressed mood have been investigated in early adulthood. We accounted for potential reverse causality in the association between depression and cognitive styles by adjusting for baseline symptoms of depression. That said, however, it remains possible that there were some individuals in the analyses who had past depression prior to 18 and who may have retained negative cognitive styles from this time, i.e., the ‘scarring hypothesis’.

Finally, the study took full account of different aspects of cognitive styles by including both the CSQ-SF and DAS-SF and by using structural equation modelling techniques to separate constructs of the CSQ-SF. The fact that the unique contribution of global attributions was only identified using latent traits further highlights the importance of this approach. Indeed, as shown in Table E1 ‘of interest’ variance accounted for only a small proportion of the total variance for each CSQ-SF item. Therefore, a high proportion of the information provided by summed scores represents nuisance variance (>70%) unrelated to global, stable and selfworth constructs specifically. This may explain why previous studies using summed scores have failed to identify the importance of global attributions. The DAS did not contain enough items across different measurement approachs to derive similar latent factors, it therefore, remains possible that a larger effect would be seen for the DAS concept if this were possible.

However some limitations should also be acknowledged. Firstly the sub-sample of ALSPAC who completed the CSQ-SF at age 18 was a relatively advantaged sub-group of young adults, meaning that the results may be less representative of higher risk populations. Indeed it is hypothesised in cognitive models of depression, that the association between cognitive styles and depression would be greater in high risk samples undergoing stress (diathesis stress). It is therefore, important to investigate the potentially moderating effect of stressful circumstances on the associations between cognitive styles and depression in future studies. It is also important to note that associations were attenuated in sensitivity analyses which extended to a larger and less advantaged sample. One possible explanation for this is that the association between depressed mood at age 18 and 19 was under-estimated in the complete case sample because those with more chronic depression were most likely to have missing data. Thus, once this association was more accurately estimated it had a greater effect on the associations between cognitive styles and later depressed mood. Nonetheless, the association between global attributions and later depression remained in this sensitivity analysis.

In addition, the follow-up was relatively short (10 months) and was within a specific time period (between 18 and 19 years of age). This period reflects the transition to adulthood. At this time changes in home, social, educational and career environments are inevitable and cognitive styles may be particularly relevant to depression (Hankin and Abramson, 1999). It is also a time of increased incidence of depression (Zalsman et al., 2006). Nonetheless, further studies are needed to compare the relative importance of global, stable and self-worth attributions in the association with depression across longer follow-up periods and at different life-stages.

### 4.2 Implications

The findings suggest that understanding the developmental origins specifically of *global* attributions may be important in preventing depression. The importance of global attributions also has implications for prevention strategies for depression where challenging beliefs of low self-worth is a core focus. The present findings suggest that concentrating on making such beliefs less global could be just as effective as attempting to remove such low self-worth beliefs entirely. This approach could provide a more focused and thus achievable target for prevention. It may be acceptable (even beneficial to realistic career/life choices) to accept that we are not good at something, as long as this does not extend to other domains. For example, we could accept that a failed job application may say something about our skills relating to that job specification, while also focusing on the fact that this bears no implications for our aptitude for other jobs, or our skills as a friend/partner. Encouragement of more specific (i.e., non-global) attributions to negative events by parents and teachers could thus potentially provide an effective prevention and treatment strategy (see case example). Finally, the findings that the scenarios accounted for a large proportion of variance on the CSQ also highlight the possible importance of the scenario in both the measurement and understanding of cognitive styles.

XXX was an 18 year old student who recently started a summer internship in a medical lab. She experienced a lot of anxiety about doing things well and reported difficulty in coping. During a course of Cognitive Behavioural Therapy a conditional belief was identified that could be summarised as “If I don׳t do things perfectly then I am a failure as a person”. She often felt stressed when she was slightly late for school and her new internship, or had forgotten things. In one incident, she had forgotten to take some extra work into school. She then had thoughts “I am hopeless” and “I can never do anything right” and her mood dropped. The therapist worked with the patient to reduce the global way she interpreted these minor things going wrong. Rather than thinking that one minor event means that everything else will go wrong, she was encouraged to treat it as an isolated incident with little influence over other events in her life or her own view of herself.

### 4.3 Conclusions

Consistent with cognitive theories of depression, the present findings provide evidence for the association between ‘depressogenic’ cognitive style and baseline and future depressed mood in a large community sample. The findings suggest that the tendency to attribute negative events to causes that are *global* could be particularly important for future depression. Reducing global attributions in response to events may thus be key in the prevention and treatment of depression, through CBT or other psychological therapies.

## Ethical standards

The authors assert that all procedures contributing to this work comply with the ethical standards of the relevant national and institutional committees on human experimentation and with the Helsinki Declaration of 1975, as revised in 2008.

## Role of funding source

The UK Medical Research Council (Grant ref: 74882) the Wellcome Trust (Grant ref: 076467) and the University of Bristol provide core support for ALSPAC. The current project was supported by a Wellcome grant held by GL (084268/Z/07/Z) and an Elizabeth Blackwell Institute for Health Research Institutional Wellcome Strategic Award supporting an early career fellowship held by Dr. Pearson. This article is the work of the authors, and Dr Pearson will serve as guarantor for the contents of this article and the analyses of data.

## Conflict of interest

None.

## Figures and Tables

**Table 1:** Confounding variables across high and low CSQ-SF total scores, based on a median split.

	High CSQ-SF score *n*=1896	Low CSQ-SF score *n*=1896	*χ*^2^
Female	1110 (59%)	1100 (56%)	*χ*^2^(1)=1.7 *P*=0.187
Social class			
(Highest) 1	165 (10%)	126 (8%)	
2	625 (38%)	600 (36%)	
3	695 (42%)	808 (49%)	*χ*^2^(1)=12.7 *P*=0.002
4	134 (8%)	112 (7%)	
(Lowest) 5	19 (1%)	19 (1%)	
Baseline depression diagnosis from CIS-R	191 (11%)	80 (4%)	*χ*^2^(1)=53.2 *P*<0.001
Maternal age	30 (5)	29 (5)	

**Table 2:** Correlation matrix and means and standard deviations for summed-score variables and latent traits (*n*=1698).

	CSQ_SF global		CSQ_SF self-worth		CSQ_SF stable	DAS-SF
CSQ_SF global	1					
CSQ_SF self-worth	Summed score	Latent	1			
0.606	**0.522**
CSQ_SF stable	Summed score	Latent	Summed score	Latent	1	
0.614	**0.663**	0.620	**0.564**
DAS-SF	0.277		0.364		0.273	1
Mean (sd)	38 (6)		36 (8)		40 (7)	22 (6)

*Note*: As can be seen, cognitive style constructs were highly correlated with each other. As would be expected DAS-SF scores show the strongest correlation with CSQ-SF self-worth attributions. Correlations between global and stable attributions increase when using latent traits compared to when using summed scores. In contrast, correlations between global and stable attributions and low self-worth are weaker when using latent traits. This would be consistent with latent traits more closely representing the theoretical constructs than summed scores, because theoretically global and stable attributions are more strongly related (and together form the basis of the hopelessness theory of depression) than attributions of low self-worth, which, more closely represent Beck׳s ‘negative’ self-beliefs and are more strongly correlated with the DAS-SF.

**Table 3:** Odds ratios to exceed thresholds for depressed mood at 19 for each 1 standard deviation increase in the CSQ-SF or DAS-SF score/sub-score at age 18.

	**Model 1**	**Model 2**	**Model 3**	**Model 4**	**Model 5**	**Model 6**	**Model 7**
**Univariable unadjusted association**	**Model 1+removed cases who are depressed at 18 using CIS-R**	**Model 2+adjusted SMFQ score at 18**	**Model 3+CSQ-SF adjusted for DAS-SF and DAS-SF adjusted for CSQ-SF**^a^	**Model 4+using MICE to impute missing data up to those with complete CSQ-SF & DAS-SF**	**Model 5+using MICE to impute missing data up to those with available predictors of CSQ-SF & DAS-SF**	**Model 6+adjusting for maternal age, social class and child sex**
***N*****=1698**	***N*****=1573**	***N*****=1573**	***N*****=1573**	***N*****=3537**	***n*****=8086**	***n*****=8086**
**CSQ-SF total score**	1.80 (1.6–2.0)	1.80 (1.6–2.0)	1.33 (1.2–1.6)	1.34 (1.15–1.6)	1.25 (1.1–1.25)	1.10(1.0–1.2)	1.08 (1.0–1.2)
	*P*<0.001	*P*<0.001	*P*<0.001	*P*<0.001	*P*=0.002	*P*=0.234	*P*=0.165
Global	1.73 (1.5–2.0)	1.68 (1.5–1.9)	1.35 (1.2–1.6)	1.30 (1.1–1.5)	1.22 (1.1–1.4)	1.12 (1.1–1.3)	1.12 (1.0–1.2)
	*P*<0.001	*P*<0.001	*P*<0.001	*P*=0.001	*P*=0.004	*P*=0.034	*P*=0.041
Stable	1.67 (1.5–1.9)	1.57 (1.4–1.8)	1.26 (1.1–1.5)	1.18 (1.0–1.4)	1.24 (1.1–1.4)	1.10 (1.0–1.3)	1.11 (1.0–1.3)
	*P*>0.001	*P*<0.001	*P*=0.005	*P*=0.052	*P*=0.003	*P*=0.147	*P*=0.095
Self-worth	1.74 (1.5–2.0)	1.67 (1.4–1.9)	1.32 (1.1–1.5)	1.25 (1.1–1.5)	1.22 (1.1–1.4)	1.02 (0.9–1.2)	1.0 (0.9–1.2)
	*P*<0.001	*P*<0.001	*P*<0.001	*P*=0.008	*P*=0.008	*P*=0.731	*P*=0.661
**Dysfunctional Attitudes Scale DAS-SF**	1.85 (1.6–2.1)	1.73 (1.5–2.0)	1.30 (1.1–1.5)	1.16 (0.9–1.4)	1.15 (1.0–1.3)	1.14 (1.0–1.3)	1.14 (1.0–1.3)
	*P*<0.001	*P*<0.001	*P*=0.001	*P*=0.088	*P*=0.082	*P*=0.037	*P*=0.033

a Adjusted for subscales of CSQ-SF.

**Table 4:** Standardized regression coefficients for latent construct factors from the CSQ-SF and the association with both baseline and future symptoms of low mood. Associations are given for factors modelled in isolation and for each construct factor mutually adjusted for all other construct factors in a combined model.

Complete case samplefor CSQ & both MFQ	**Baseline mood**		**Future mood**		**Future mood adjusted for baseline mood**	
*N*=1888	Standardized regression coefficient representing: association between each factor and SMFQ symptoms at age 18		Standardized regression coefficient representing: association between each factor and SMFQ symptoms at age 19		Standardized regression coefficient representing: association between each factor and SMFQ symptoms at age 19, adjusted for MFQ at age 18	
**Univariable association**	**Adjusted for other factors**	**Univariable association**	**Adjusted for other factors**	**Univariable association**	**Adjusted for other factors**
**Global factor (age 18)**	0.54 (95% CI 0.4–0.6)	0.44 (95% CI 0.3–0.6)	0.42 (95% CI 0.4–0.5)	0.37 (95% CI 0.2–0.5)	0.13 (0.1–0.2)	0.20 (95% CI 0.1–0.3)
*P*<0.001	*P*<0.001	*P*<0.001	*P*<0.001	*P*<0.001	*P*=0.005
**Self**-**worth factor (age 18)**	0.43 (95% CI 0.4–0.5)	0.18 (95% CI 0.01–0.27)	0.30 (95% CI 0.3–0.4)	0.14 (95% CI 0.1–0.2)	0.04 (−0.01 to 0.1)	0.01(95% CI −0.01 to 0.08)
*P*<0.001	*P*<0.001	*P*<0.001	*P*<0.001	*P*=0.143	*P*=0.723
**Stable factor (age 18)**	0.46 (95% CI 0.4–0.5)	0.13 (95% CI −0.01 to 0.27)	0.35 (95% CI 0.3–0.4)	0.16 (95% CI 0.02–0.3)	0.08 (0.02–0.13)	(95% CI −0.1 to 0.1)
*P*<0.001	*P*=0.062	*P*<0.001	*P*<0.001	*P*=0.012	*P*=0.460
