# Supplementary material for: Cognitive styles and future depressed mood in early adulthood: The importance of global attributions
Source: J Affect Disord. 2015 Jan 15;171:60–7. doi: 10.1016/j.jad.2014.08.057 (PMC4222738; doi:10.1016/j.jad.2014.08.057)
Supplement: Supplementary file 1 — Supplementary data [file mmc1.pdf]

Table E1: Proportion of variance of each CSQ-SF item that was attributable to latent construct factors, scenario factors and unexplained variance (uniqueness). Combined 'nuisance' variance is highlighted.

		Scenario A	B	C	D	F	G	H	I
Stability	Construct of interest	13.3%	26.1%	40.8%	22.0%	27.7%	20.4%	11.2%	11.0%
	Scenario	6.8%	27.2%	12.4%	23.3%	25.5%	28.4%	1.2%	14.7%
	Uniqueness	80.2%	47.2%	46.2%	55.4%	47.8%	51.5%	87.7%	74.4%
Self	Construct of interest	27.0%	41.8%	53.6%	34.7%	29.8%	24.7%	32.8%	18.9%
	Scenario	12.0%	11.1%	11.0%	30.0%	24.9%	18.7%	23.6%	22.1%
	Uniqueness	60.7%	46.8%	35.9%	35.7%	45.5%	57.1%	43.2%	59.0%
Globality	Construct of interest	13.0%	15.8%	33.1%	12.4%	6.3%	8.8%	18.6%	18.2%
	Scenario	32.5%	27.7%	0.8%	47.5%	8.6%	33.3%	39.3%	13.7%
	Uniqueness	54.6%	56.6%	66.0%	40.5%	85.2%	58.1%	42.5%	68.2%
